# Antibacterial activity of *pelargonium graveolens* essential oil nanoemulsion evaluated by microfluidics and DESI mass spectrometry

**DOI:** 10.1038/s41598-026-49846-9

**Published:** 2026-04-23

**Authors:** Zinab Moradi Alvand, Fateme Aghamir, Kimia Torabi, Liana Parseghian, Alireza Ghassempour, Mohammad Hossein Mirjalili, Hasan Rafati

**Affiliations:** 1 Department of Phytochemistry, Medicinal Plants and Drugs Research Institute, Shahid Beheshti University, Tehran, Iran; 2 Department of Agriculture, Medicinal Plants and Drugs Research Institute, Shahid Beheshti University, Tehran, Iran; 3 Department of Pharmaceutical Engineering, Medicinal Plants and Drugs Research Institute, Shahid Beheshti University, Tehran, Iran

**Keywords:** Nanoemulsion, *Pelargonium graveolens*, Essential oil, Desorption Electrospray Ionization, Microfluidic system, Antibacterial activity, Biochemistry, Biological techniques, Biotechnology, Microbiology, Nanoscience and technology

## Abstract

Ensuring microbial safety in pharmaceutical applications remains a critical public health priority, motivating the development of novel antimicrobial delivery systems targeting pathogenic microorganisms. The antibacterial activity of a nanoemulsion formulated with *Pelargonium graveolens* essential oil (*P. graveolens* EO) was evaluated against two bacterial species, *E. coli* (ATCC 25922) and *B. subtilis* (ATCC 23857), using both conventional and microfluidic techniques. The optimized *P. graveolens* essential oil nanoemulsion exhibited a mean droplet size of 68.94± 3.1 nm, with surfactant, essential oil, and HLB values optimized at 5.29% w/w, 2.60% w/w, and 11.82, respectively. The effects of the nanoemulsion on bacterial cells were assessed by monitoring the release of intracellular proteins, nucleic acids, and potassium ions, as well as by determining the minimum inhibitory concentration (MIC), minimum bactericidal concentration (MBC), and conducting time-kill assays. The use of microfluidic technology significantly enhanced bacterial exposure to the nanoemulsion due to increased contact areas between bacterial membranes and nanodroplets. With microfluidic methods, the MIC values for *B. subtilis* and *E. coli* were determined within 15 min, compared to 3.12 and 6.25 µg/mL over 24 h by conventional means. Notably, *B. subtilis* exhibited greater resistance to the nanoemulsion than *E. coli*, likely due to differences in their cell wall structures. Further analysis using desorption electrospray ionization mass spectrometry revealed changes in the bacterial cell membrane, particularly through the detection of released phosphatidylglycerol and phosphatidylethanolamine. These results indicate that the nanoemulsion prompted more pronounced structural alterations in Gram-negative *E. coli* cells. These results highlight the potential of *P. graveolens* EO nanoemulsions as innovative antimicrobial agents for pharmaceutical use.

**Supplementary Information:**

The online version contains supplementary material available at 10.1038/s41598-026-49846-9.

## Introduction

*Pelargonium graveolens* is a medicinal plant highly valued for its diverse pharmacological activities. Both its extract and essential oil are recognized for their antimicrobial, antifungal, anti-inflammatory, and hypoglycemic effects^1,2^. The essential oil (EO) of *P. graveolens* is rich in bioactive compounds such as geraniol, citronellol, and linalool, which are primarily responsible for its potent antibacterial activity^3^. Given these broad pharmacological properties, research continues to further investigate the antibacterial potential of EO and to elucidate its qualitative and quantitative chemical composition^4^. However, it should be noted that, like most essential oils, *P. graveolens* EO is physiologically unstable and sensitive to temperature, light, and oxygen, and generally exhibits low water solubility^1,5^. EO is highly regarded in the field of medicine due to its broad spectrum of pharmacological properties. Rich in bioactive constituents, EO demonstrates potent antimicrobial, antifungal, anti-inflammatory, and hypoglycemic activities, making it a promising natural agent for therapeutic use^1,3^. Recent studies have reported that *P. graveolens* EO exhibits notable inhibitory effects against various Candida strains and displays pronounced antibacterial potency toward both Gram-positive and Gram-negative bacteria, underlining its potential for the therapy of infectious diseases^3,6^. Additionally, the high antioxidant content of *P. graveolens* EO can be beneficial in mitigating oxidative stress-related disorders by neutralizing free radicals^4^. The essential oil has also demonstrated promising results in the management of diabetes, owing to its hypoglycemic effects, as well as in the alleviation of chronic inflammation^7^. However, it is important to note that, like most essential oils, EO is physiologically unstable and generally exhibits low water solubility, factors that may influence its pharmaceutical formulation and storage^8^. These considerations are vital for developing effective and stable medicinal products based on EO.

To maximize their effectiveness, nano-drug delivery systems have been used more and more. This may be because of their larger surface-to-volume ratio, which increases their contact with cell membranes, enhancing their solubility and bioavailability while also protecting and regulating their release^9,10^. Because of their easy manufacturing and desired functional qualities, nanoemulsions are more appropriate for usage in pharmaceutical products among the nanoparticle systems currently used to transport bioactive components^11^. Compared to synthetic ones, the use of natural antimicrobial substances in the creation of nanoparticles can offer several benefits. Their longer-lasting stability is most likely the cause of this^12^. This disrupts the bacterial membrane and results from an electrostatic interaction between nanoparticles^13^. Consequently, the bacterial membrane is destroyed by nanoparticles. Three primary constituents of bacterial membranes include phospholipids, peptides, and carbohydrates, which can react with nanoparticles to degrade both the cell wall and the cell membrane^14^.

Research has extensively investigated the antibacterial properties of EOs derived from various plants. Studies have demonstrated that natural components make a substantial contribution to enhancing the antibacterial activity of EO-based nanoemulsions. For instance^15^, highlighted the potent antibacterial properties of *Satureja khuzistanica* EO, which is rich in carvacrol. This EO showed activity against bacterial strains such as *Salmonella typhi*, *E. coli*, *S. aureus*, *S. dysenteriae*, and *K. pneumoniae* when incorporated into hydroxypropyl methylcellulose edible films containing *Thymus daenensis* EO nanoemulsion^16^. Moreover^17^, investigated the antibacterial effects of *Satureja khuzistanica* EO on *E. coli* using microfluidic chips, while^18^ evaluated the effectiveness of *Thymus daenensis* EO nanoemulsion against *S. pneumoniae*,* P. aeruginosa*, and *H. influenzae*. Recent studies^19^ have reported the development of *Satureja khuzistanica* EO NE, emphasizing their promising physicochemical properties and potential pharmaceutical applications. These nanoemulsions demonstrated enhanced antibacterial activity against *S. aureus* compared to standard *Satureja khuzistanica* EO nanoemulsions, signifying their potential for improved applications in both pharmaceutical formulations and medical safety. Since the early 1990 s, when the promising Lab-on-a-chip (LOC) technology was founded on the microstructure’s platform, microfluidic devices have produced notable benefits for the study of mammalian cells^20^. Microfluidics systems have matured during the past ten years, resulting in several applications in engineering and biological sciences^21^. Rapid mass/heat transfer is facilitated by enormous surface area per unit volume, careful control of both time and spatial parameters with considerable sensitivity and productivity, minimal input of reagents and materials, and low-cost, long-lasting bacterial sites, combined with separate production and operating processes are some of its unique benefits over traditional approaches resulting in enhanced biological responses in line with rapid analysis^22^. Additionally, time-saving assays and quick assessments of the interactions between materials and/or living cells are significant benefits of microfluidic devices^23^.

This article examines the antibacterial properties of *P. graveolens* EO nanoemulsion (*P. graveolens* EO NE) against both Gram-positive and Gram-negative bacteria, utilizing conventional techniques and innovative microfluidic technologies. A microfluidic device was developed to study the inhibitory process, focusing on key indicators such as protein concentration, nucleic acid release, and potassium leakage resulting from bacterial cell breakdown at varying nanoemulsion concentrations and flow rates (residence times). The bacterial microstructure and morphological changes were qualitatively evaluated using optical microscopy and scanning electron microscopy (SEM). Additionally, Desorption Electrospray Ionization (DESI) mass spectrometry provided insights into chemical changes in biological samples and the mechanism of bacterial cell membrane degradation without the need for extensive sample preparation.

## Materials and methods

### Extraction of essential oil

The extraction of essential oil was carried out using the hydrodistillation method with a Clevenger-type apparatus^19^. Briefly, the air-dried plant material was ground into a coarse powder, and an appropriate amount (100 g) was placed in a round-bottom flask containing distilled water at a plant material-to-water ratio sufficient to allow complete immersion.

The mixture was subjected to hydrodistillation for 5 h using the Clevenger apparatus. During the distillation process, the essential oil was separated from the aqueous phase and collected. The obtained essential oil was dried over anhydrous sodium sulfate (Na₂SO₄) to remove residual moisture. The extracted oil was stored in dark glass vials at 4 °C until further analysis.

### Design of experiment

Design of experiments (DOE) for optimizing technological processes is widely recognized. Among the various DOE approaches, the Box-Behnken design (BBD) and the central composite design (CCD)-the latter being a form of response surface methodology (RSM)-are particularly popular among researchers. Based on preliminary tests, the following variable ranges were determined to be suitable for the three process variables: HLB values of 8, 10, and 12; EO concentrations of 1.0%, 2.0%, and 3.0% (w/w); and surfactant concentrations of 3.0%, 4.5%, and 6.0% (w/w). To properly evaluate experimental error, a total of 17 experiments were designed according to the BBD, including 5 replicates at the center point (Table S1). The association between droplet size in nanoemulsions and these parameters was then analyzed using the quadratic regression model given in Eq. (1).

1 $$\:Y={\beta\:}_{0}+\:{\beta\:}_{1\:}{X}_{1}+\:{\beta\:}_{2\:}{X}_{2}+\:{\beta\:}_{3\:}{X}_{3}+\:{\beta\:}_{12\:}{X}_{1}{X}_{2}+\:{\beta\:}_{13\:}{X}_{1}{X}_{3}+\:{\beta\:}_{23\:}{X}_{2}{X}_{3}+\:{\beta\:}_{11}{X}_{1}^{2}\:+\:{\beta\:}_{22}{X}_{2}^{2}\:+\:{\beta\:}_{33}{X}_{3}^{2}$$

In this analysis, Y, representing the droplet size (nm), is modeled using a regression equation where β₀ serves as the intercept coefficient, β₁, β₂, and β₃ represent the linear coefficients, β₁₁, β₂₂, and β₃₃ denote the quadratic coefficients, and β₁₂, β₁₃, and β₂₃ capture the interaction effects between variables; the experimental data supporting this model were processed using Design-Expert software (version 7.0.0, State-Ease Inc.).

### Development and stability analysis of *P. graveolens* EO NE

Seeds of *P. graveolens* (lot code: PG-M25-0324) were purchased from Behkesht Co. (Karaj, Iran). The seeds were sown in the greenhouse of Shahid Beheshti University in mid-March 2025, and essential oil extraction was performed after full vegetative growth following the supplier’s standard cultivation protocol. A voucher specimen of *P. graveolens* was authenticated and deposited in the Herbarium of Yasuj University under voucher number 1016 (YUH).

The *P. graveolens* EO NE was prepared as described in the protocol by^24^ with minor modifications. The preparation process involved adding a Tween 80 and water mixture to the pre-emulsified blend of *P. graveolens* EO and Span 80 under continuous stirring to ensure uniform mixing. The emulsion was then subjected to a high-speed homogenization process using a homogenizer set at 16,000 rpm for 20 min to achieve a fine and stable droplet size distribution. The stability of the nanoemulsions was evaluated over a three-month storage period by monitoring the variation in average droplet size under refrigerated conditions (4 ± 1 °C), ensuring the assessment of long-term size stability.

### Fabrication of microfluidics and experimental setup

The microchannel was fabricated using a microlithography technique based on SU-8 photolithography followed by soft lithography, with minor modifications to optimize channel performance, as adapted from Ref^17^. Briefly, a silicon wafer was cleaned and spin-coated with SU-8 photoresist to define the channel height. After soft baking, the photoresist was exposed to ultraviolet (UV) light through a photomask, followed by post-exposure baking and development to obtain a negative-relief SU-8 master mold. The resulting microchannel depth was approximately 150 μm.

Polydimethylsiloxane (PDMS, Sylgard 184; Dow Corning, USA) was prepared at a 10:1 (w/w) base-to-curing-agent ratio, degassed, poured onto the SU-8 mold, and cured at 90 °C for 30 min. The cured PDMS replica was peeled off, and inlet and outlet ports were created using a 1.25 mm diameter biopsy punch. The PDMS layer was then irreversibly bonded to a flat PDMS substrate via oxygen plasma treatment (8 mbar, 40 W, 1 min) to form enclosed microchannels.

The device comprises three inlet channels and one outlet. Each inlet channel is 150 μm wide with a length of 7 mm, merging into a main channel with a width of 300 μm and a straight length of approximately 3.3 cm. The main channel includes alternating straight and curved sections to enhance mixing, with straight segment lengths ranging from 10 mm and an outer curvature radius of 0.50 mm, ensuring smooth flow transitions and stable hydrodynamic conditions.

## Antibacterial activity

### MIC and MBC assay methods

The antibacterial activity of the samples was evaluated through the broth dilution technique to establish the minimum inhibitory concentration (MIC), which is defined as the lowest concentration that prevents visible microbial growth. This procedure was conducted in accordance with the method described by^25^, wherein the samples were serially diluted in Mueller-Hinton Broth (MHB) in 96-well plates, resulting in final concentrations ranging from 0.012 to 25 µg/mL for the nanoemulsion, and 0.78 to 1600 µg/mL for the essential oil. The MBC, which represents the minimum concentration required to eliminate bacterial populations (confirmed by the absence of visible colonies on agar plates), was determined using the spot inoculation method described by^26^. For this, 100 µL of inoculum from wells with no visible microbial growth was spread onto Mueller-Hinton Agar (MHA) plates and incubated at 37 °C for 24 h. To ensure reliability, all MIC and MBC assays were conducted in triplicate, with the average values recorded.

### Well-diffusion assay

The antibacterial properties of *P. graveolens* were assessed using the agar well-diffusion method, following the protocol outlined by^27^. In this assay, 100 µL of bacterial suspension was uniformly spread onto MHA plates to achieve consistent bacterial coverage. After the plates were aseptically dried, 6 mm wells were made in the agar using a sterile Pasteur pipette. Each well was carefully filled with 50 µL of the test solution. The plates were then incubated at 37 °C for 24 h to permit evaluation of bacterial inhibition. Gentamicin (15 µg/well) served as positive control, while DMSO was employed as a negative control to confirm the absence of antimicrobial effects from the solvent. Antibacterial activity was quantified by measuring the diameters of the inhibition zones surrounding each well. All experiments were conducted in triplicate, and mean values were reported to ensure result reliability and consistency.

### Assessment of membrane disruption

To assess the release of nucleic acids, proteins, and potassium ions, cultures of *E coli* and *B. subtilis* were grown in MHB and adjusted to an OD_600_ of 0.4–0.6 using a spectrophotometer to ensure uniform bacterial density. The bacterial suspensions were then exposed to the *P. graveolens* EO NE at final concentrations of 3.12 µg/mL for *E. coli* and 6.25 µg/mL for *B. subtilis*. Each treated sample (100 µL) was incubated at 37 °C with constant shaking at 250 rpm for 2 h to investigate the effect of the nanoemulsion on bacterial membrane integrity. After incubation, the suspensions were spun at 4000 rpm for 10 min to pellet bacterial cells, and the resulting supernatant, containing molecules and ions released due to membrane disruption, was collected for analysis. The number of nucleic acids released was measured by recording absorbance at 260 nm, while protein concentration was determined at 280 nm using a UV-visible spectrophotometer. Potassium ion concentrations were analyzed using atomic absorption spectrometry, with a standard curve generated from potassium solutions ranging from 1.5 to 10.0 ppm (1.5, 2.0, 2.5, 5.0, 7.0, and 10.0 ppm) for accurate quantification.

To enhance the conventional approach, supplementary samples were collected from a microfluidic system every 15 min under MIC conditions. The samples were spun at 4000 rpm for 10 min and analyzed using spectrophotometry and atomic absorption spectrometry, following the methodology described by^17^. The microfluidic system enabled real-time, dynamic observation of biomolecular and ionic alterations induced by the *P. graveolens* EO nanoemulsion, providing a more detailed understanding of its antimicrobial properties.

### Evaluation of time-kill and growth inhibition

The time-kill assay was performed to evaluate the survival of *B. subtilis* and *E. coli* in the presence of EO and *P. graveolens* EO NE. Bacterial suspensions were prepared by adjusting the cell density to 4.5–5.5 × 10⁵ CFU/mL in MHB and were added to tubes containing sterilized compounds at their respective MICs. The tubes were maintained at 37 °C, and bacterial viability was evaluated by plating samples collected at 20, 40, 60, 80, 100, and 120 min intervals. The control group consisted of bacterial suspensions in MHB without any treatment to track natural bacterial growth.

Furthermore, 5 mL of the bacterial suspension was introduced into a microfluidic chip preloaded with the nanoemulsion at its MIC concentration, enabling precise and controlled interaction between the bacteria and the antimicrobial agent.

The bacteria were incubated in the chip for the designated residence time under controlled conditions. Serial dilutions of the bacterial samples were prepared using 0.085% standard saline solution to ensure countable colonies. To evaluate colony-forming units (CFU), 100 µL of the diluted samples was plated onto nutrient agar and incubated at 37 °C for a period of 24 h. After incubation, colonies were counted to quantify bacterial survival^28^.

### Bacteria morphology

SEM was used to observe morphological changes on the bacterial cell surface after treatment with *P. graveolens* EO (NE) for 15 min within a microfluidic system. Following treatment, bacterial cells were preserved by fixing them overnight at 4 °C with 2.5% glutaraldehyde to maintain their structural integrity. The cells were subsequently washed twice with sterile phosphate-buffered saline (PBS) to remove any remaining fixative and impurities. To minimize structural damage from sudden solvent transitions, gradual dehydration was carried out using a graded ethanol series (60%, 70%, 80%, 90%, and 100%), with each concentration applied for 10 min. Following dehydration, the samples were covered with a 10 nm gold layer using a sputter coater to improve electrical conductivity for imaging. The gold-coated samples were subsequently analyzed under SEM with an accelerating voltage of 5–20 kV to examine surface morphology and detect structural modifications resulting from *P. graveolens* EO NE treatment^29^.

### Mass spectrum analysis using DESI

The DESI technique offers an advanced approach for quick and direct analysis of condensed-phase samples in ambient conditions, eliminating the need for complex sample preparation or the laborious task of introducing the sample into the mass spectrometer’s vacuum system^30^.

For bacterial sample preparation, 50 µL of bacterial suspension was transferred into a sterile microtube, where it was standardized to a 0.5 McFarland concentration (approximately 4.5–5.5 × 10⁵ CFU/mL) using LB medium to ensure uniform bacterial density; subsequently, 125 µL of methanol and 62.5 µL of chloroform were carefully introduced to the suspension, which was then subjected to vigorous vortexing for 5 min to facilitate efficient lipid extraction from the bacterial cells.

After this, an additional 62.5 µL of chloroform was added to enhance lipid extraction, followed by another 1 min of vortexing. The sample was centrifuged at 9,000 rpm for 10 min, allowing the separation of a lipid- and fatty-acid-containing supernatant.

The supernatant was carefully transferred onto a clean microscope slide and left to air dry at room temperature, ensuring minimal disturbance to the sample’s integrity. For bacterial samples subjected to nanoemulsion treatment through the microfluidic system, the processed output was meticulously collected and centrifuged at 9,000 rpm for 10 min to concentrate the material, after which lipids were extracted and dried using the same standardized protocol. Dried lipid samples from untreated and treated bacterial suspensions were analyzed directly using DESI-MS to assess lipid composition and potential alterations caused by the nanoemulsion treatment.

### DNA extraction method

Genomic DNA was extracted from E. coli suspensions using a column-based purification protocol. Briefly, three microtubes containing bacterial suspensions were centrifuged at 4,000 rpm for 5 min to pellet the cells. The supernatant was discarded, and each pellet was resuspended in 500 µL of pre-digestion buffer supplemented with 10 µL of Proteinase K solution. The mixtures were vortexed thoroughly to ensure complete homogenization and incubated at 55 °C for 30 min to allow enzymatic digestion.

Following the initial digestion step, 400 µL of digestion buffer was added to each tube and mixed by vortexing for 20 s. Subsequently, 300 µL of precipitating solution was introduced, and the samples were vortexed briefly at high speed for 5 s to facilitate precipitation of unwanted cellular components. The lysates were then carefully transferred to column filters placed in sterile collection microtubes and centrifuged at 13,000 rpm for 1 min to separate the clarified solution from cellular debris and lipid fractions.

The columns were washed with 400 µL of wash buffer 1 and centrifuged at 13,000 rpm for 1 min. The flow-through was discarded to remove residual contaminants. A second washing step was performed using 400 µL of wash buffer 2, followed by centrifugation at 13,000 rpm for 1 min. This washing procedure was repeated to ensure complete removal of impurities.

After the final wash, the columns were transferred to fresh, sterile microtubes. Purified DNA was eluted by adding 90 µL of solubilizing buffer directly onto the column membrane, followed by incubation at 65 °C for 3–5 min. A final centrifugation step at 13,000 rpm for 1 min was performed to collect the eluted DNA, thereby completing the extraction procedure and yielding purified bacterial DNA^31,32^.

### Method of preparing agarose gel for electrophoresis

A 1% (w/v) agarose gel was prepared by dissolving 0.3 g of agarose in 30 mL of 1× TAE buffer. The mixture was heated until complete dissolution of the agarose. After cooling to 50–60 °C, ethidium bromide was added to a final concentration of approximately 0.5 µg/mL. The gel solution was poured into a casting tray fitted with a comb and allowed to solidify at room temperature prior to electrophoresis.

### DNA breakage investigation by compounds

To evaluate DNA breakage, 4 µL of genomic DNA was mixed with 4 µL of nanoemulsion at three different concentrations, including MIC, below MIC (< MIC), and above MIC (> MIC). As a positive control, 4 µL of DNA was mixed with 4 µL of 30% hydrogen peroxide. The mixtures were loaded onto a pre-prepared 0.8–1% agarose gel and subjected to electrophoresis at 80–100 V for 45–60 min. DNA fragmentation was visualized by ethidium bromide staining and documented using a gel documentation system under UV illumination^33^.

## Results and Discussion

### Essential oil composition

The essential oil components were analyzed using the highly accurate and sensitive gas chromatography-mass spectrometry (GC-MS) method, as detailed in Table 1. The essential oil derived from *P. graveolens* was analyzed using GC-FID and GC-MS approaches. A total of 27 components were detected. The major constituents of the oil included citronellol (36.68%), geraniol (15.36%), linalool (11.68%), citronellyl butyrate (7.35%), and citronellyl isobutyrate (5.63%). The characteristic GC-MS chromatogram of the essential oil of *P. graveolens* in Fig. S1. As reported by^34^, the notable concentrations of citronellol and the principal bioactive components identified in *P. graveolens* EO significantly contribute to its potent antibacterial activity.

**Table 1:** Chemical composition of the EO Jamzad.

No.	Compounds	Percent (%)
1	Citronellol	36.68
2	Beta-pinene	0.36
3	Geraniol	15.36
4	1-Octanol,2,7-dimethyl	1.50
5	Phthalic acid, di(2-propylpentyl)-ester	1.71
6	α-phellandrene	0.05
7	Germacrene D	0.37
8	p-cymene	0.12
9	Geranyl formate	0.06
10	Citronellyl butyrate	7.35
11	Geranyl isobutyrate	0.60
12	Citronellyl tiglate	0.07
13	beta-Phenylethyl butyrate	0.57
14	Glutaric acid, 3-methylbut-2-en-1-yl geranyl ester	0.20
15	o-Menth-8-ene	0.10
16	Citronellyl valerate	0.23
17	alpha-Terpineol	0.62
18	Citronellyl isobutyrate	5.63
19	trans-β-caryophyllene	0.09
20	Geranyl acetate	0.75
21	Geranyl isovalerate	1.25
22	Glutaric acid, 3-methylbut-2-yl neryl ester	0.13
23	Guaiol	2.80
24	D- Limonene	2.96
	Linalool	11.68
26	6-Octen-1-ol, 3,7-dimethyl-formate	8.23
27	Phenylethyl Alcohol	0.53

^*^RI: Retention indices relative to C9-C22 n-alkanes on the DB-5 column.

### Optimization of nanoemulsion formulation

To optimize and stabilize the droplet size of the nanoemulsion, the Box-Behnken Design (BBD), a statistical technique under response surface methodology (RSM), was utilized. The droplet size, chosen as the primary response variable, was measured using the dynamic light scattering (DLS) method, a reliable and accurate approach for determining droplet size in colloidal systems. The optimization process involved varying three crucial independent factors: surfactant concentration (A), essential oil concentration (B), and hydrophilic-lipophilic balance (HLB), as detailed in Table S1.

The mean droplet size was determined by averaging three independent DLS measurements to ensure precision and consistency. The relationships between droplet size and the independent variables were modeled using quadratic regression equations, provided in both actual and coded variable forms (Eqs. 2 and 3). These equations were instrumental in predicting the optimal conditions required to achieve a stable nanoemulsion with minimized droplet size.

2 $$\begin{aligned}&\:Response\:Drplet\:size\:of\:nanoemulsion\left(\:Actual\right)=Mean\:droplet\:size=\\&-95.69000+29.55167\times\:\mathrm{S}\mathrm{u}\mathrm{r}\mathrm{f}\mathrm{a}\mathrm{c}\mathrm{t}\mathrm{a}\mathrm{n}\mathrm{t}+76.58500\times\:\mathrm{E}\:\mathrm{O}+13.21875\times\:\mathrm{H}\mathrm{L}\mathrm{B}\\&-4.18333\times\:\mathrm{S}\mathrm{u}\mathrm{r}\mathrm{f}\mathrm{a}\mathrm{c}\mathrm{t}\mathrm{a}\mathrm{n}\mathrm{t}\times\:\:\mathrm{O}+1.76667\times\:\mathrm{s}\mathrm{u}\mathrm{r}\mathrm{f}\mathrm{a}\mathrm{c}\mathrm{t}\mathrm{a}\mathrm{n}\mathrm{t}\times\:\mathrm{H}\mathrm{L}\mathrm{B}-2.76250\times\:\mathrm{E}\:\mathrm{O}\:\times\:\mathrm{H}\mathrm{L}\mathrm{B}\\&-4.89556\times\:{\mathrm{S}\mathrm{u}\mathrm{r}\mathrm{f}\mathrm{a}\mathrm{c}\mathrm{t}\mathrm{a}\mathrm{n}\mathrm{t}}^{2}-5.74000\times\:{\mathrm{E}\:\mathrm{O}}^{2}-1.00375\times\:{\mathrm{H}\mathrm{L}\mathrm{B}}^{2}\end{aligned}$$

$$\:Respnse:Droplet\:Size\:of\:nanoemulsion\:\left(Coded\right)=Mean\:droplet\:size$$

3 $$\:=+86.78-7.81\times\:A+7.18\times\:\mathrm{B}-8.86\times\:\mathrm{C}-6.28\times\:\mathrm{A}\times\:\mathrm{B}+5.30\times\:\mathrm{A}\times\:\mathrm{C}-5.53\times\:\mathrm{B}\times\:\mathrm{C}+\:-11.02\times\:{A}^{2}-5.47\times\:{B}^{2}-4.01\times\:{C}^{2}$$

The outcomes of the quadratic response surface model’s ANOVA are summarized in Table 2. The statistical relevance of each coefficient is reflected by the P-value, while the model’s robustness is demonstrated by the notably high F-value (F = 67.12) along with the extremely low P-value (*p* < 0.0001). Additionally, the Lack of Fit test was not significant (*p* = 0.0690) when compared to the pure error, suggesting that the model adequately represents the observed experimental data. The quality of the model was also assessed by the coefficient of determination (R²), which was found to be 0.9885, demonstrating a strong correlation between experimental and predicted values. Additional key statistical metrics for the model include: a standard deviation of 2.12, a coefficient of variation (C.V.%) of 2.75, an adjusted R² of 0.9738, adequate precision of 23.550, and a predicted R² of 0.8496, all within statistically acceptable ranges. Overall, plotting response surface curves and predicting optimal stability conditions confirmed that the fitted model is suitable for optimizing the system’s stability.

**Table 2:** ANOVA results for independent variables obtained from RSM.

Source	Sum of squares	Degree of freedom	Mean square	F-value	*p*-value Prob > F
Model	2703.54	9	300.39	67.12	<0.0001 *significant.*
*A-Surfactant*	488.28	1	488.28	109.09	<0.0001
B-Essential Oil	411.85	1	411.85	92.02	<0.0001
C-HLB	628.35	1	628.35	140.39	<0.0001
AB	157.50	1	157.50	35.19	0.0006
AC	112.36	1	112.36	25.10	0.0015
BC	122.10	1	122.10	27.28	0.0012
A^2^	510.86	1	510.86	114.14	<0.0001
B^2^	138.73	1	138.73	30.99	0.0008
C^2^	67.87	1	67.87	15.16	0.0059
Residual	31.33	7	4.48		
Lack of ଁt	25.10	3	8.37	5.37	0.0690 is *not significant.*
Pure error	6.23	4	1.56		
Core total	2734.87	16			

Fig. S2A presents a plot comparing the actual and predicted results. This plot is useful for identifying any data points that the regression model cannot accurately predict, and it also shows how closely the experimental and predicted values correspond. Fig. S2B displays the residuals plotted against the predicted response values. This plot is used to check for constant variance in the residuals; the points should be randomly scattered across the plot. The observed random distribution of residuals confirms the reliability of the proposed model. Additionally, Fig. S2C shows a normal probability plot of the residuals, illustrating the difference between the observed data and the predicted values. For a normal distribution, the data points should fall along a straight line. The pattern observed in this plot further supports that the residuals are normally distributed, confirming the model’s validity.

### The combined influence of formulation factors on the droplet size of the nanoemulsion

**Fig. 1:**
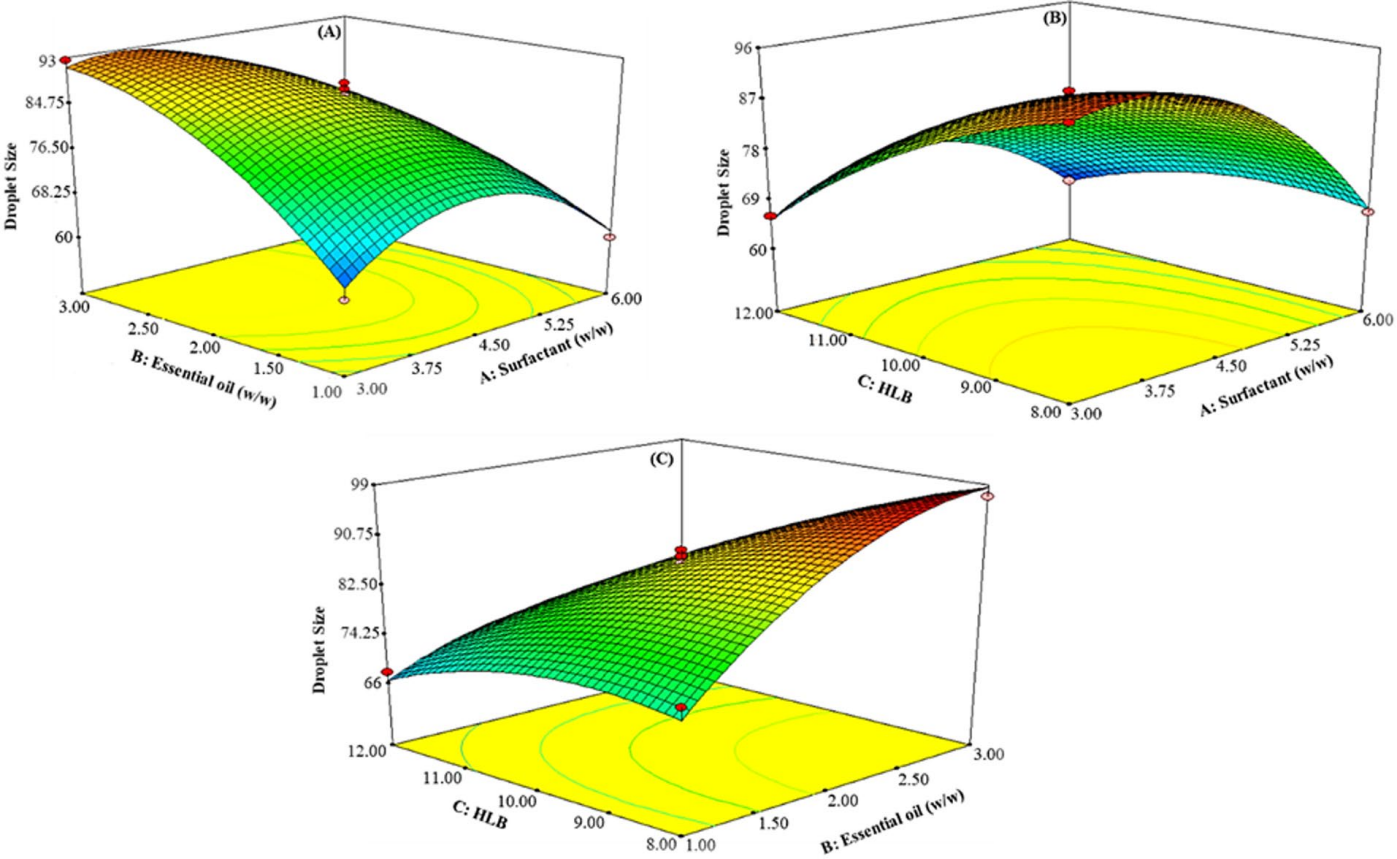
Interaction effect of (A) surfactant and EO, (B) surfactant and HLB, and (C) HLB and EO on the droplet size.

The size of droplets in nanoemulsions largely depends on the proportions of their ingredients. Although higher surfactant levels can effectively minimize droplet size, they may also elevate toxicity risks in pharmaceutical formulations^35,36^. To determine the optimal droplet size, the proportions of EO, surfactant concentration, and HLB value were systematically varied. Tween 80 and Span 80 were selected as the surfactants because of their low toxicity, mild irritancy, high stability, and effectiveness in forming nanoemulsions with small droplet sizes. Figure 1 displays three-dimensional response surface diagrams generated using quadratic polynomial equations, illustrating how the independent variables interact to affect droplet size. The interplay between essential oil and surfactant concentrations in influencing droplet size is illustrated in Fig. 1A. The droplet size initially decreases as EO and surfactant concentrations increase, but gradually increases at higher levels. The smallest droplet sizes were observed when both EO and surfactant concentrations were at moderate levels, indicating this range as optimal. These findings align with previous studies, such as^37^, which reported a similar trend in droplet size reduction with increased surfactant concentration in cinnamon oil nanoemulsions.

Figure 1B demonstrates how varying the concentrations of surfactant and HLB impacts the droplet size of the nanoemulsion. The results indicate that increasing the HLB value and surfactant concentration reduces droplet size, reaching a minimum point where the smallest droplets are observed. This effect occurs because surfactants lower the free energy needed to form nanoemulsions; their hydrophilic and hydrophobic components help reduce surface tension at the water-oil interface, bridging the gap between the two phases^38,39^. Therefore, increasing Tween 80 concentration generally results in smaller nanoemulsion droplets. Figure 1C illustrates how the EO and HLB values jointly influence the average droplet size. The saddle-shaped curve in this figure implies a significant interaction between HLB and EO levels. Additionally, the p-values provided in Table 2 confirm the statistical significance of these process variables.

### Droplet size optimization

**Fig. 2:**
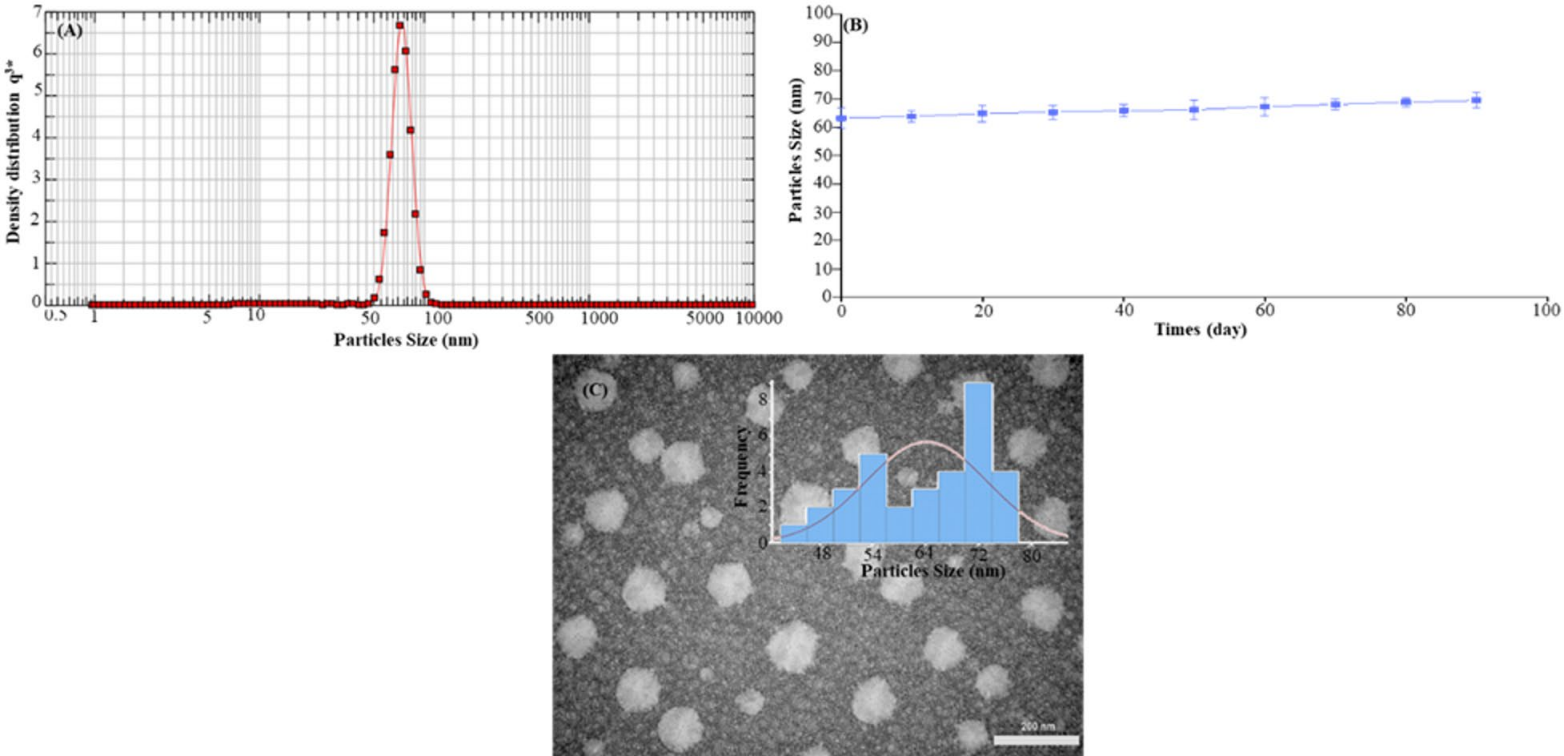
Average droplet size distribution of the nanoemulsion determined by DLS (A), stability after 80 days (B), and TEM images with number-frequency histograms (C).

The regression model was utilized to determine the optimal settings for the chosen variables. To validate the accuracy of this model, one separate case study were performed (Table 3). The resulting nanoemulsions had an average droplet size of 68.94 ± 3.1 nm, as determined by DLS measurements (Fig. 2A). Assessments after 80 days of storage at 4 °C showed no significant change in average droplet size, indicating that the nanoemulsion possess long-term storage stability (Fig. 2B). After 80 days, the polydispersity index (PDI) remained low at 0.23, suggesting a uniform size distribution. In addition, the measured zeta potential (− 11.8 mV) demonstrates sufficient electrostatic repulsion among droplets, reinforcing the stability of the formulated nanoemulsion system. In addition to DLS measurements, Transmission Electron Microscopy (TEM) images (Philips-CM30) were used to visualize the nanoemulsions under optimal conditions (Fig. 2C). The TEM images confirmed that the droplets were spherical and uniformly sized. Furthermore, the number frequency histograms in Fig. 2C highlight the size distribution of the nanoemulsion droplets, with a predominant size of 64 nm on a linear scale.

**Table 3:** The optimal point selected for the validation of nanoemulsion.

No.	Surfactant A (w/w %)	Essential Oil B (w/w %)	HLB C	Predicted Droplet size (nm)	Reported Droplet size (nm)	Desirability
1	5.29	2.60	11.82	68.06	68.94 nm	1.000

### MIC and MBC

The MIC and MBC for EO and NE against the *E. coli* and *B. subtilis* bacteria are shown in Tables 4 and 5. Nanoemulsion has a much greater level of antibacterial activity than the EO sample. According to Tables 4 and 5. NE MIC and MBC against *E. coli* were found to be 3.12 µg/mL, and EO MIC and MBC against *E. coli* were found to be 200 µg/mL and 400 µg/mL. Nanoemulsion MIC and MBC against *B. subtilis* were found to be 6.25 µg/mL and 12.50 µg/mL, respectively, and EO MIC and MBC against *B. subtilis* were found to be 800 µg/mL and 800 µg/mL, respectively. A high surface-to-volume ratio nanoemulsion treatment increased the permeability of active chemicals within the cell membrane, which in turn caused more damage. A rapid microfluidic device intensifies this effect^30^.

**Table 4:** MIC and MBC values of *P. graveolens* EO against bacteria.

Bacteria	MIC (µg/mL)	MBC (µg/mL)
*E. coli*	200	400
*B. subtilis*	800	800

**Table 5:** MIC and MBC values of *P. graveolens* EO NE against bacteria.

Bacteria	MIC (µg/mL)	MBC (µg/mL)
*E. coli*	3.12	3.12
*B. subtilis*	6.25	12.50

Furthermore, the antibacterial activity of the NE and EO against Gram-positive and Gram-negative bacteria was assessed using the agar diffusion method, as illustrated in Fig. S3A, B. The findings indicate that *E. coli* (Gram-negative) and *B. subtilis* (Gram-positive) were susceptible to the nanoemulsion, evidenced by the inhibition zones observed at different sample concentrations. Similarly, raising the initial sample concentration (6.25–25.0 µg/mL) can improve the corresponding inhibitory zone, which supports the observations. The findings imply that the manufactured nanoemulsion has more potent antibacterial qualities than the EO by itself.

#### Time-Dependent Bacterial Inhibition

Fig. S4 demonstrates that sample concentration affects the maximum inhibition of *E. coli* and *B. subtilis* bacteria after 15 min. Fig. S4 shows that nanoemulsion significantly inhibits bacterial growth (control OD_600_ = 0.450), and at the MICs, nearly all bacteria are killed after 15 min within the microfluidic device.

### Membrane Integrity and Antibacterial Efficacy

The release of proteins and nucleic acids from treated bacterial cells serves as an indicator of membrane damage, since detecting these molecules in the surrounding medium reflects disrupted membrane integrity. When cells are damaged, they release intracellular substances such as proteins into their environment.

**Fig. 3:**
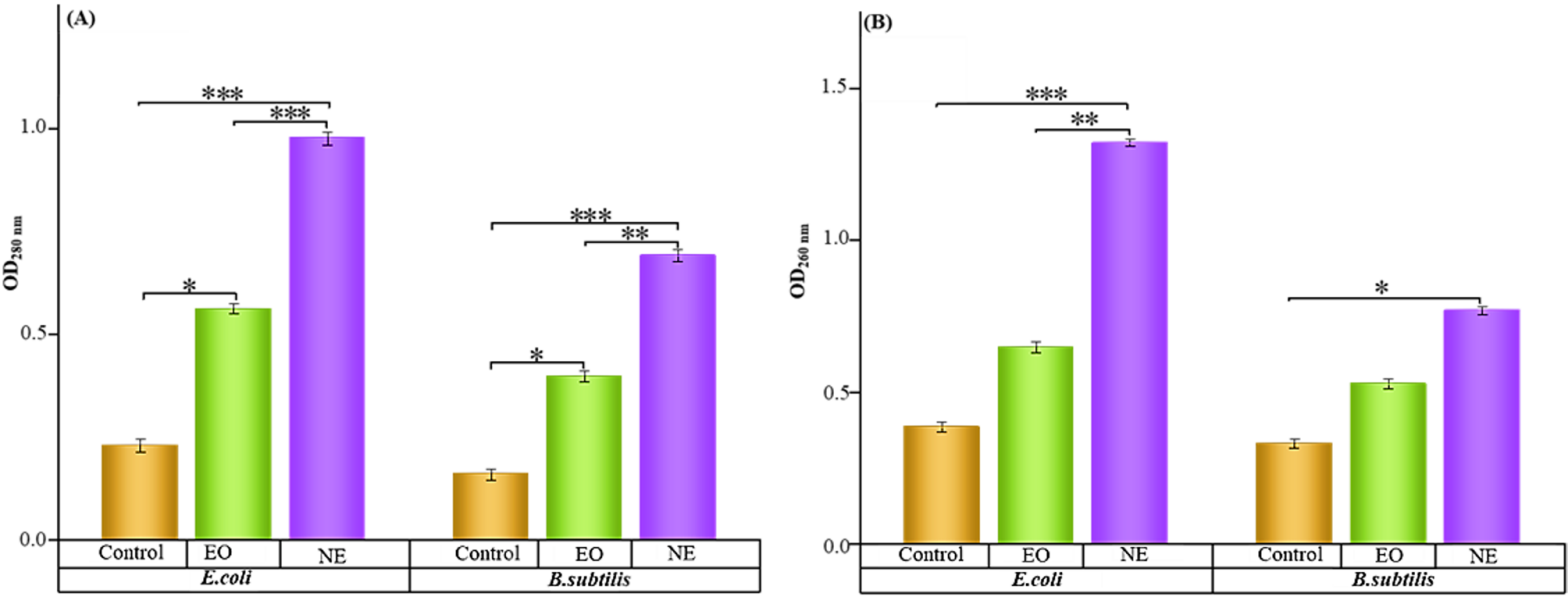
Protein and nucleic acid release (A, B) from *E. coli* and *B. subtilis* bacteria strains were treated 15 min in a microfluidic system at MIC concentration.

To evaluate the antibacterial activity of the nanoemulsion against *E. coli* and *B. subtilis*, the leakage of intracellular proteins and nucleic acids was measured by absorbance at 280 nm and 260 nm, respectively, using both traditional methods and a microfluidic chip (Table S2 and Fig. 3).

For nucleic acids (OD_260_), treatment with EO and NE at MIC levels caused an increase in absorbance for *E. coli* from 0.365 to 0.573 (EO) and 1.07 (NE), while for *B. subtilis* it rose from 0.380 to 0.485 (EO) and 0.687 (NE) within 2 h compared to controls (Table S2, S3). For proteins (OD_280_), absorbance values increased from 0.270 to 0.70 (EO) and 0.921 (NE) for *E. coli*, and from 0.15 to 0.442 (EO) and 0.629 (NE) for *B. subtilis* within the same period. In the microfluidic system, nucleic acid absorbance (OD_260_) increased within 15 min of incubation, rising from 0.385 to 0.647 (EO) and 1.32 (NE) for *E. coli*, and from 0.330 to 0.527 (EO) and 0.769 (NE) for *B. subtilis* relative to the control (Fig. 3B).

Protein absorbance (OD_280_) increased after 15 min, changing from 0.230 to 0.732 (EO) and 0.967 (NE) for *E. coli*, and from 0.160 to 0.495 (EO) and 0.692 (NE) for *B. subtilis* compared to controls (Fig. 3A).

This can be explained by the high surface area-to-volume ratio in the microfluidic system, which allows surface forces, such as surface tension, to dominate. These forces reduce body and inertial effects while enhancing the interaction between the bacterial cell membrane and the active components^40^.

#### Cytoplasmic membrane disruption and potassium efflux

The breakdown of the cell membrane, a sign of nanoemulsion action, results in potassium release. Potassium leakage was measured using atomic absorption spectrophotometry with a 766.5 nm potassium hollow cathode lamp, a 0.5 nm bandpass filter, and an air-acetylene flame. Table 6 shows that potassium leakage from *B. subtilis* and *E. coli* bacteria, treated with the substance at the MIC for 2 h, increased significantly. Control groups had potassium levels of 0.37 ± 0.005 ppm and 0.35 ± 0.007 ppm, but after treatment, these levels increased to 2.80 ± 0.031 ppm and 1.40 ppm for *E. coli* and *B. subtilis*, respectively.

**Fig. 4:**
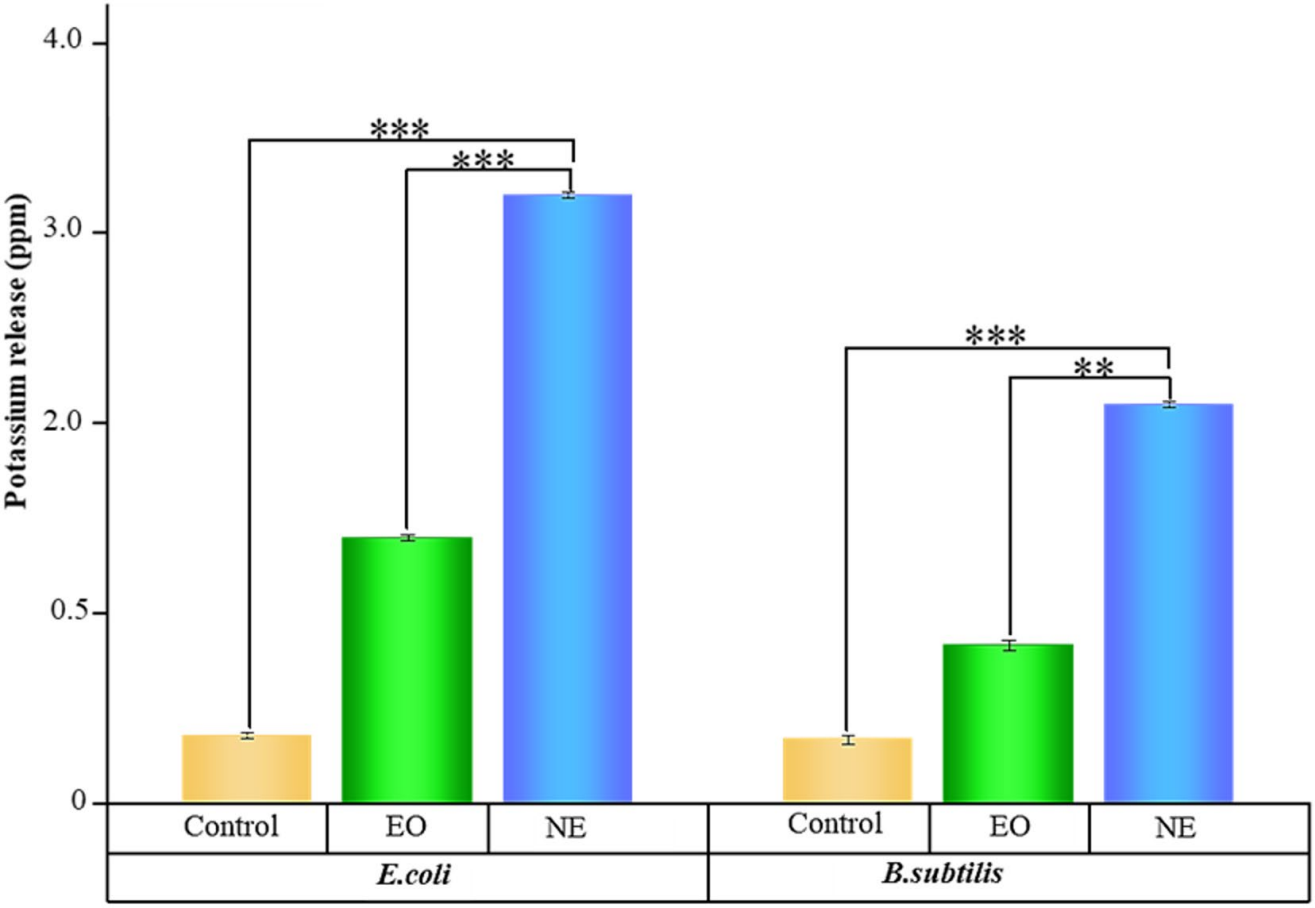
Potassium release from *E. coli* and *B. subtilis* at the MIC concentration of the nanoemulsion after 15 min of exposure. Based on the data analysis using one-way ANOVA, a significant change was found (*p* < 0.01*) compared to the control group.

The potassium leakage in bacteria treated with the nanoemulsion was significantly higher than in the control group (*p* < 0.01). Potassium levels in the control group remained nearly constant at 0.36 ± 0.01 ppm for *B. subtilis* and 0.34 ± 0.03 ppm for *E. coli* (Fig. 4). However, after 15 min of treatment, potassium concentrations rose to 3.25 ± 0.05 ppm and 2.10 ± 0.05 ppm for *B. subtilis* and *E. coli*, respectively. These findings align with previous studies^17,30,41^, suggesting that microfluidic chips effectively suppress bacterial growth within a short period.

It should be mentioned that some of the bacteria in the control group may have been killed during their regular life cycle, which could be connected to the potassium content in that group^26^. After the cells were exposed to the MIC concentration of EO and NE for 15 min, intracellular contents were released. This suggests a change in the permeability of the cytoplasmic membrane. Gram-positive bacteria released less K^+^ than Gram-negative bacteria (*p* < 0.05).

**Table 6:** Measurements of potassium leakage (ppm) from *E. coli* and *B. subtilis* for 2 h of treatment at their MICs.

Bacteria	*E. coli*	*B. subtilis*
Control	0.37 ± 0.005	0.35 ± 0.007
EO	1.20 ± 0.047	0.875 ± 0.082
NE	2.80 ± 0.031	1.40 ± 0.064

#### Reduction of bacterial viability

**Fig. 5:**
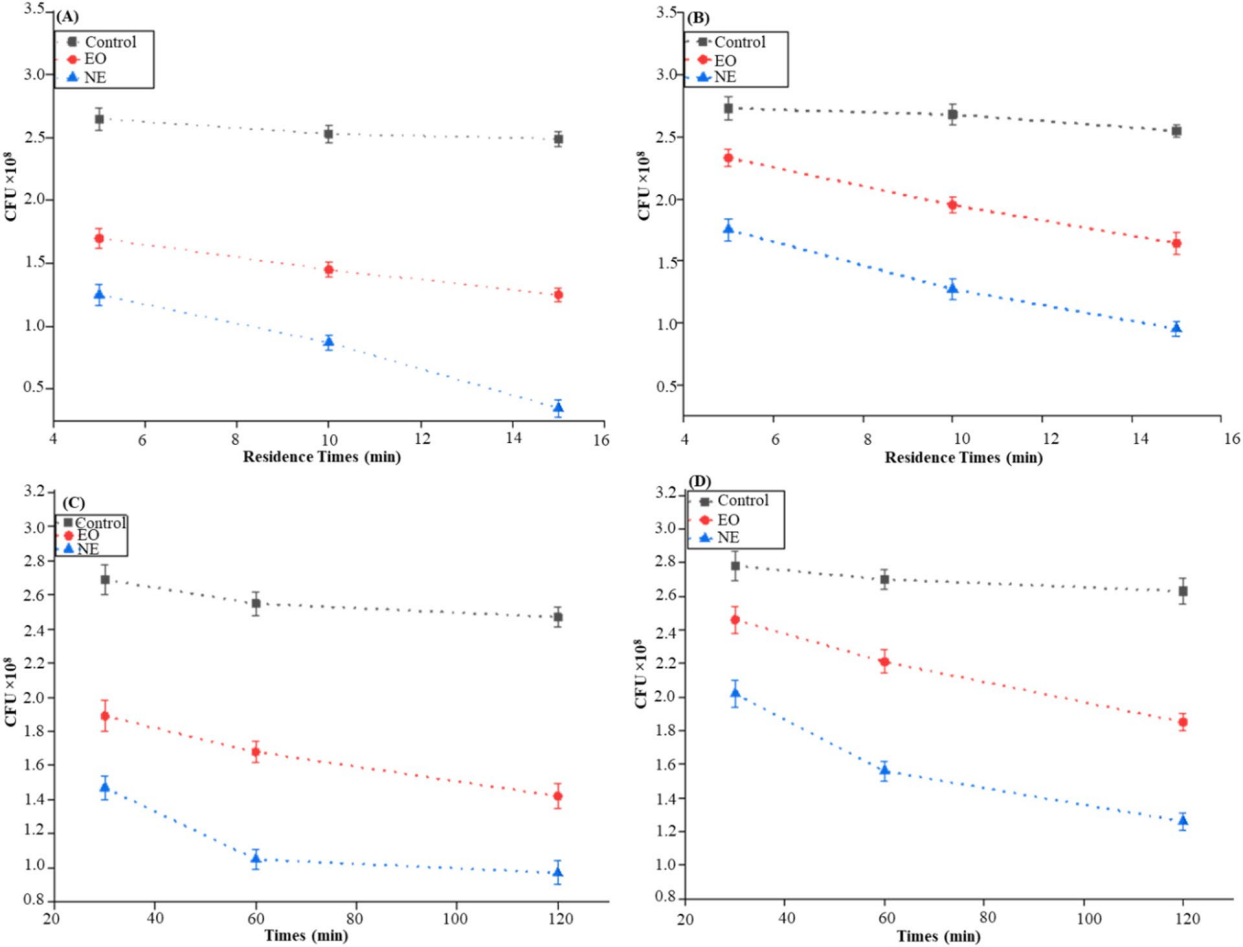
Time-killing assay of *E. coli* and *B. subtilis* cells after treatment, (A, B) by the microfluidic system at MIC concentration, and (C, D) conventional technique at MIC concentration.

Figure 5A–D illustrate the patterns of inactivation of *B. subtilis and E. coli* using standard starting cell suspensions ranging from 10⁹ to 10¹⁰ CFU/mL, treated using the conventional method and microfluidic technology. The viable cell counts steadily decreased over varying residence times, as shown in the figures. Notably, the number of bacterial colonies in Fig. 5A, B was significantly lower (*p* < 0.05) compared to the control group. Additionally, more *B. subtilis* colonies remained than *E. coli* colonies (*p* < 0.05) when compared to the control group.

#### Disruption of cell membrane integrity

**Fig. 6:**
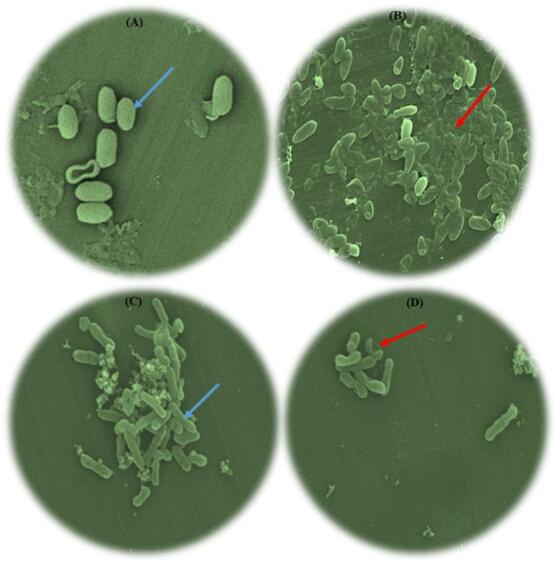
SEM images of *E. coli* and *B. subtilis* bacterial cells treated with MIC concentration *P. graveolens* EO nanoemulsion inside the microfluidic system. (A, Control) B) treated 15 min for *E. coli.* (C Control, D) treated at 15 min for *B. subtilis*, respectively.

Morphological changes in *B. subtilis* and *E. coli* were analyzed using SEM. Figure 6A–D show SEM images of bacterial cells before and after treatment with a nanoemulsion (MIC) for 15 min. Untreated cells of *B. subtilis* and *E. coli* (Fig. 6A and C) display smooth, intact cell walls with a typical rod shape, while blue arrows in these figures point to the intact cell membranes.

The red arrows in Fig. 6B and D illustrate bacterial membrane lysis and surface contraction caused by nanoemulsion adherence to the cell surfaces of *B. subtilis* and *E. coli*. This interaction induced cellular contraction and the simultaneous release of intracellular components. Structural damage was more pronounced in *E. coli* after 15 min of treatment with the nanoemulsion (Fig. 6B and D), while *B. subtilis* exhibited comparatively less membrane damage. These findings indicate that the primary antibacterial mechanism involves disrupting membrane integrity. The active compounds in EO, particularly Citronellol (36.68%) and Geraniol (15.36%), compromised the cell membranes of both bacteria, leading to increased permeability, surface defects, and morphological changes. Consistent with these observations, a previous study^30^ has shown that such membrane damage can produce ghost cells.

The removal of lipopolysaccharide (LPS) molecules in Gram-negative bacteria is associated with the substitution of phospholipids, which are essential structural elements of the bacterial outer membrane’s double-layer. This change compromises the membrane’s stability, increases its permeability, and results in membrane shrinkage^42^. One proposed mechanism for the antibacterial action of essential oils (EOs) is their interaction with these phospholipids, disrupting the membrane structure and preventing it from preserving its form. This disruption leads to leakage of cellular contents and eventual membrane rupture^43^. The resulting membrane damage causes the cells to shrink and lyse, a change that presents as ghost cell-shaped *E. coli* under morphological observation, also known as spheroplasts. Some of these damaged cells are visible, while others are only partially observed. In our earlier research, we demonstrated that the nanoemulsion affected the bacterial cell wall by breaking down LPS and enhancing the release of glycerol-3-phosphate fatty acid esters, including phosphatidyl ethanolamine (PE) and phosphatidyl glycerol (PG), as confirmed by DESI-MS analysis^30^.

### Mass spectrometry evidence

**Fig. 7:**
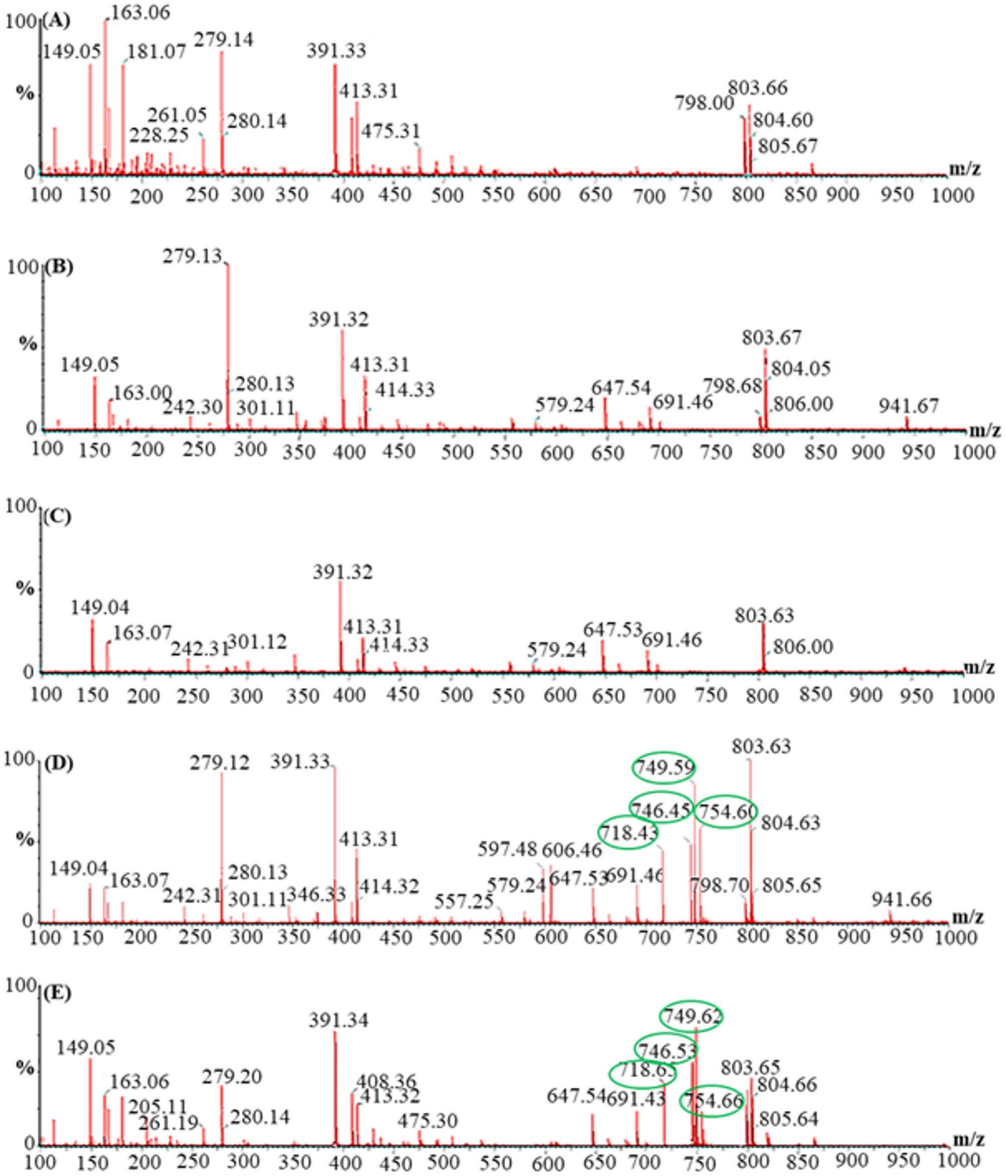
Desorption electrospray ionization (DESI) mass spectrometry (MS) related to *E. coli*, (A) Blank, (B) intact *E. coli* bacteria, (C) culture media (D) *E. coli* bacteria treated with methanol/chloroform. (E) *E. coli* bacteria treated with nanoemulsion at the MIC concentration.

To provide additional verification of bacterial cell wall disruption via detection of specific lipid biomarkers, DESI mass spectrometry analysis was performed. Figure 7 presents the positive ion mode mass spectra for *E. coli*, allowing comparison across various treatment groups: the blank (culture medium containing nanoemulsion), untreated bacteria, culture medium alone, bacteria exposed to methanol/chloroform, and bacteria treated with the minimum inhibitory concentration (MIC) of the nanoemulsion, corresponding to Fig. 7A and E, respectively. The purpose of these analyses was to distinguish primary mass spectral peaks that arise from the disruption of the bacterial membrane by the nanoemulsion, separating them from recurrent signals associated with the nanoemulsion itself (for instance, m/z 181.07 and m/z 475.30, which appear in Figs. 7A–D, are attributed to the nanoemulsion). PE and PG, which together make up the bulk of the lipid content of *E. coli* membranes, approximately 70–80% PE, 20–25% PG, and less than 5% cardiolipin, as described by^43^ were examined in detail. Notably, the peaks observed in Fig. 7E, such as PG (34:1) at m/z 749.5905, PE (37:4) at m/z 754.5962, PE (34:1) at m/z 718.4301, and PE (36:1) at m/z 746.4475, were identified as derivatives of PG and PE with reference to the LMSD database, suggesting that treatment with the EO-based nanoemulsion caused cell lysis. These results are in agreement with previous findings^44^. Furthermore, analogous experiments conducted with *B. subtilis* (Fig. S5) demonstrated that this bacterium exhibits greater resistance to the nanoemulsion treatment.

### DNA integrity analysis

**Fig. 8:**
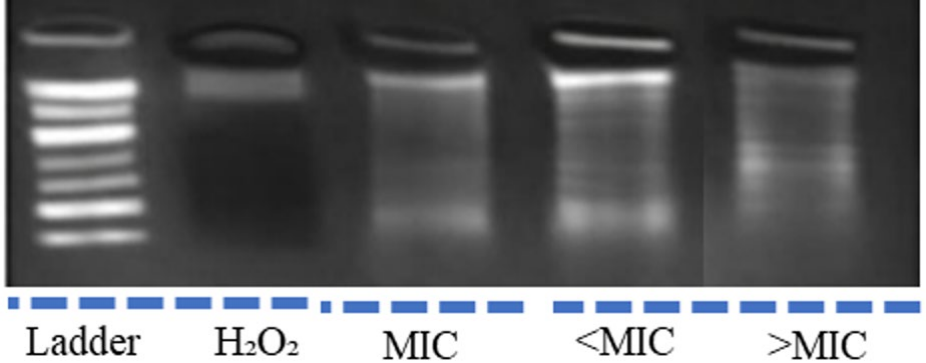
DNA damage by nanoemulsions prepared using the essential oil of *Pelargonium graveolens*.

Figure 8 and Fig. S6 shows the agarose gel electrophoresis profile of genomic DNA following treatment with nanoemulsion at different concentrations. The first lane (Ladder) represents the molecular weight marker. The lane corresponding to hydrogen peroxide (H₂O₂, positive control) exhibits pronounced smearing, indicating substantial DNA fragmentation likely due to oxidative damage.

DNA treated with nanoemulsion at concentrations below MIC (< MIC) displayed relatively intact bands with minimal smearing, suggesting limited DNA damage at sub-inhibitory levels. At MIC, slight reductions in band intensity and mild smearing were observed, indicating possible concentration-dependent effects. At concentrations above MIC (> MIC), increased smearing and decreased band intensity were evident compared to lower concentrations, suggesting enhanced DNA destabilization. These findings indicate a concentration-dependent impact of the nanoemulsion on DNA integrity.

## Conclusion

This research employed traditional as well as highly efficient microfluidic methods to examine the interaction of nanoemulsion with bacteria. It was observed that using appropriate bioactive compounds and increasing the contact surface area through nanoemulsification can play a key role in inhibiting the activity of both Gram-positive and Gram-negative bacteria. Cell lysis could result from a reaction between the naturally active chemicals and the phospholipid bilayer that makes up bacterial cells. A rapid release of cytoplasmic components, including proteins, potassium ions, and nucleic acids, would follow from the lysed cell wall’s total or partial removal. The outcome showed that, in both the conventional method and the microfluidic system, *B. subtilis* was less susceptible to the nanoemulsion treatment than *E. coli*. In contrast to the conventional method, the microfluidic system performed better in terms of a shorter residence time (15 min vs. 120 min). Additionally, the SEM imaging provided a clear illustration of the cell wall disintegration for *B. subtilis* and *E. coli*, which was shown by the shrinkage of the cell membrane surface. These findings suggest that nanoemulsions containing EO, particularly when optimized and processed using advanced microfluidic techniques, could hold significant potential for the formulation of novel antimicrobial drugs designed to treat infections from both Gram-negative and Gram-positive bacteria.

## Electronic Supplementary Material

Below is the link to the electronic supplementary material.

Supplementary Material 1

## Data Availability

All data generated or analyzed during this study are included in this article. Further enquiries can be directed to the corresponding author.
