# Supplementary material for: Antibacterial activity of pelargonium graveolens essential oil nanoemulsion evaluated by microfluidics and DESI mass spectrometry
Source: Sci Rep. 2026 Apr 23;16:18802. doi: 10.1038/s41598-026-49846-9 (PMC13273082; doi:10.1038/s41598-026-49846-9)
Supplement: Supplementary file 1 — Supplementary Material 1 [file 41598_2026_49846_MOESM1_ESM.docx]

**Supporting Information**

**Table S1**. Box–Behnken design plan, experimental and predicted results

| Run |  | Factors |  |  | Response |  |  |
| --- | --- | --- | --- | --- | --- | --- | --- |
|  | Surfactant (w/w %) A | Essential oil (w/w %) B | HLB  C |  | Droplet size  (nm) |  | Predicted |
| 1 | 4.50 | 2.00 | 10.00 |  | 85.60 |  | 86.78 |
| 2 | 4.50 | 2.00 | 10.00 |  | 88.60 |  | 86.78 |
| 3 | 4.50 | 1.00 | 12.00 |  | 67.90 |  | 66.51 |
| 4 | 4.50 | 2.00 | 10.00 |  | 87.50 |  | 86.78 |
| 5 | 6.00 | 1.00 | 10.00 |  | 60.00 |  | 61.31 |
| 6 | 4.50 | 2.00 | 10.00 |  | 86.30 |  | 86.78 |
| 7 | 3.00 | 3.00 | 10.00 |  | 92.60 |  | 91.29 |
| 8 | 6.00 | 2.00 | 12.00 |  | 60.3 |  | 60.37 |
| 9 | 3.00 | 2.00 | 12.00 |  | 66.10 |  | 65.40 |
| 10 | 6.00 | 2.00 | 8.00 |  | 66.80 |  | 67.50 |
| 11 | 3.00 | 2.00 | 8.00 |  | 93.80 |  | 93.72 |
| 12 | 4.50 | 1.00 | 8.00 |  | 75.20 |  | 73.19 |
| 13 | 6.00 | 3.00 | 10.00 |  | 65.20 |  | 63.11 |
| 14 | 4.50 | 3.00 | 12.00 |  | 67.80 |  | 69.81 |
| 15 | 4.50 | 3.00 | 8.00 |  | 97.20 |  | 98.59 |
| 16 | 4.50 | 2.00 | 10.00 |  | 85.90 |  | 86.70 |
| 17 | 3.00 | 1.00 | 10.00 |  | 62.30 |  | 64.39 |

**
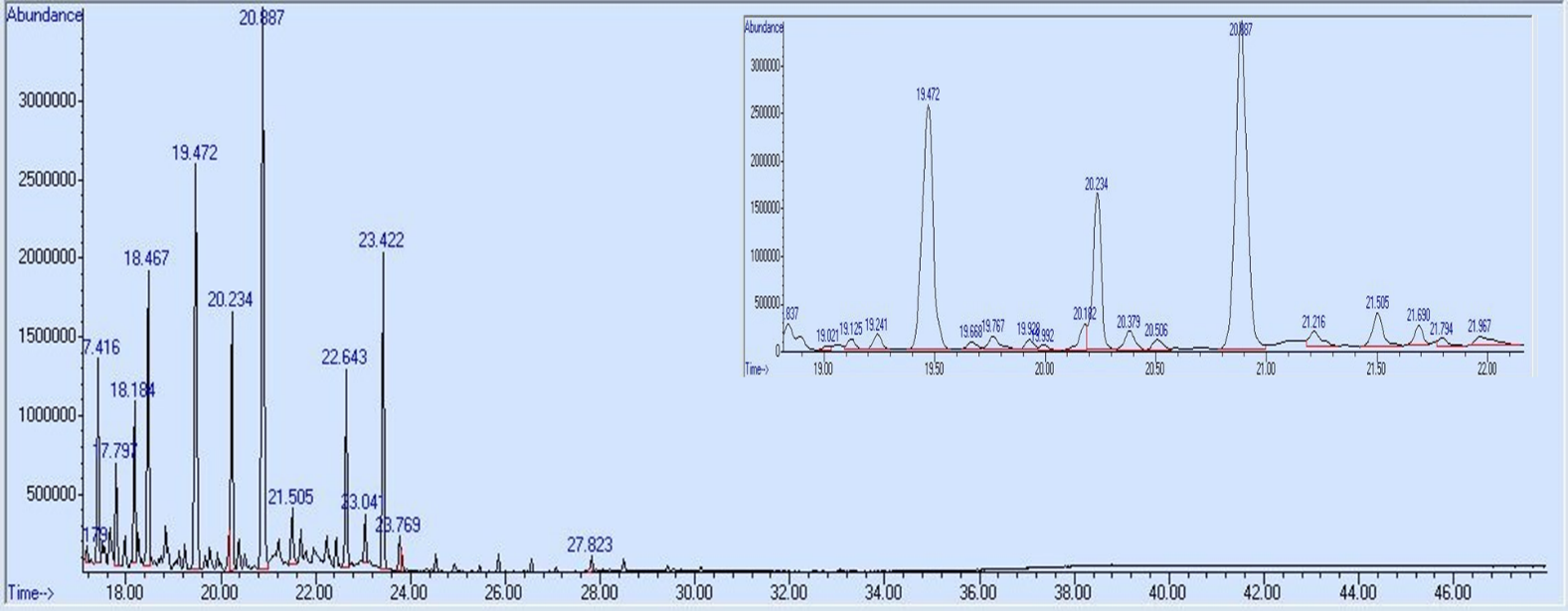
**

**Fig.S1.** GC–MS chromatogram of the essential oil of Pelargonium graveolens.

| **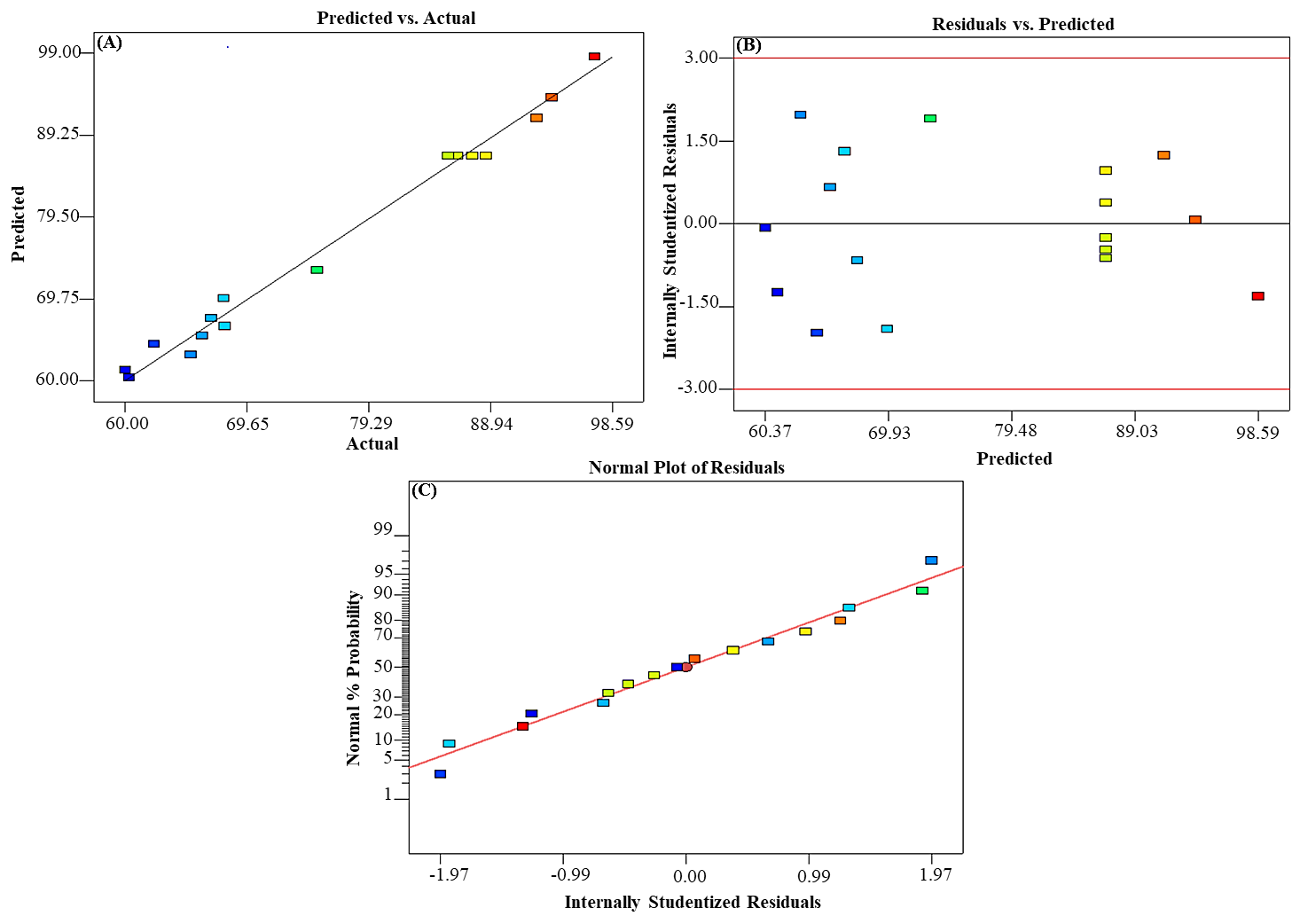** |
| --- |
| **Fig. S2.** Evaluation of RSM proposed model between predicted and experimental values; A) Predicted versus actual values plot, B) Residual versus predicted plot, C) Normal plot of residuals. |

| 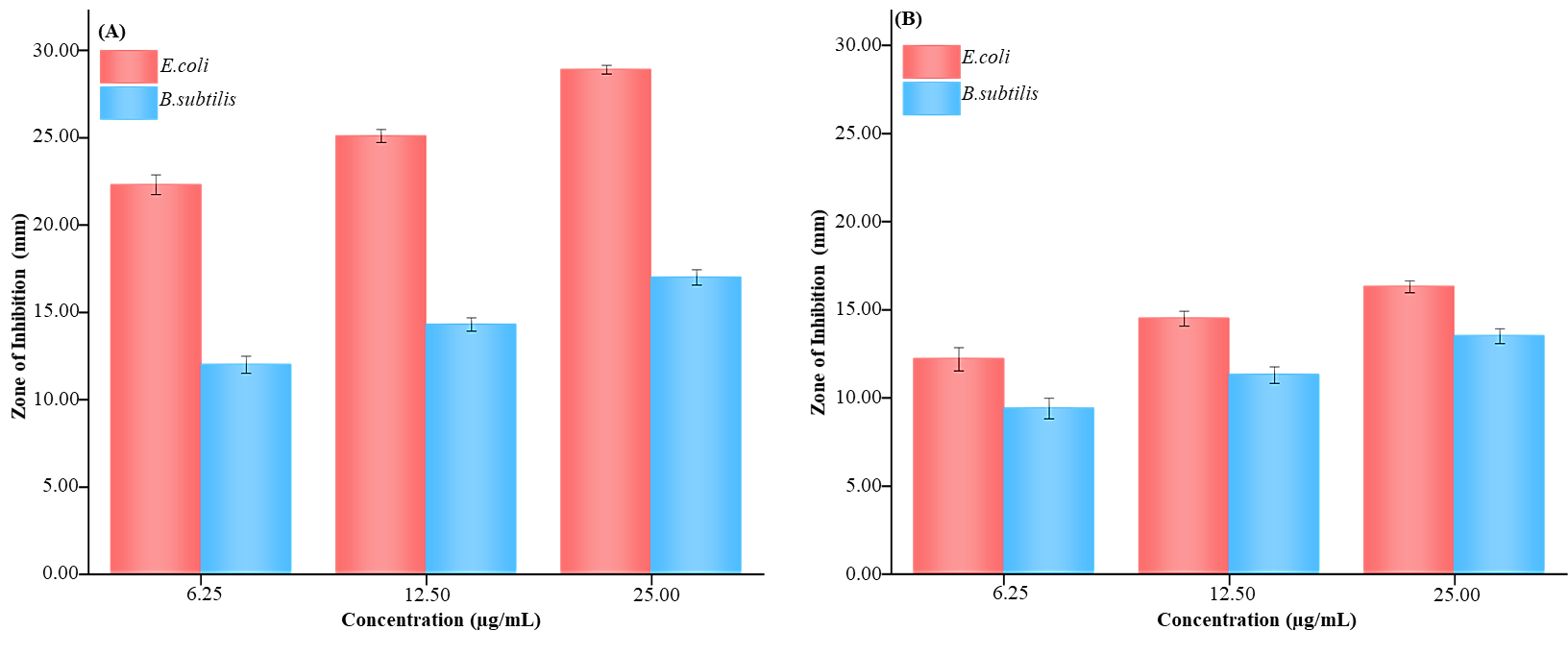 |
| --- |
| **Fig.S3.** The result of antibacterial studies by well diffusion method for (A) *P. graveolens* EO NE and (B) *P. graveolens* EO. |

| 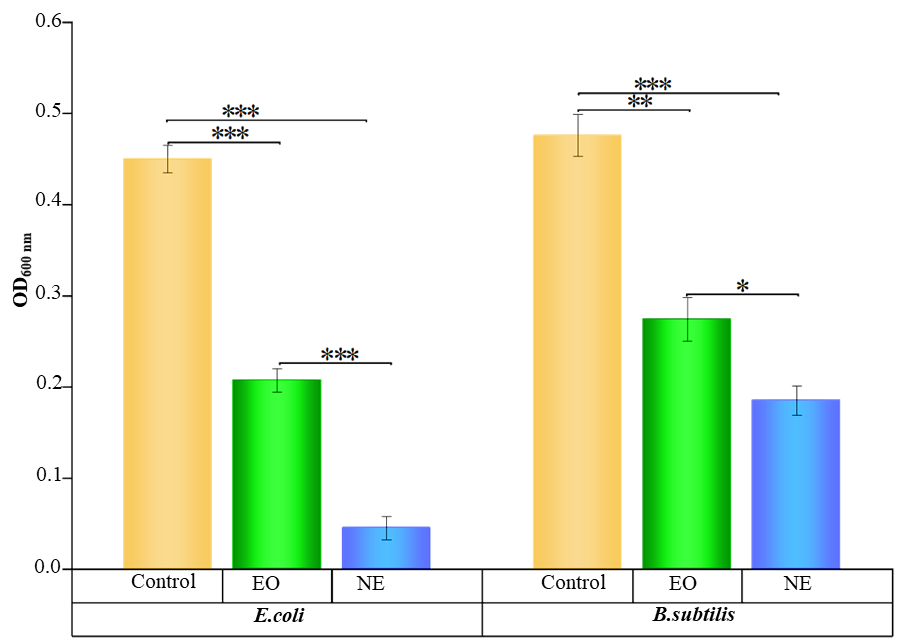 |
| --- |
| **Fig.S4.** Viability of *E. coli* and *B. subtilis* at the MIC concentrations of EO and its NE following 15 min of exposure. Statistical analysis using one-way ANOVA indicates significant differences compared with the control groups (p < 0.01 and p < 0.05). |

**Table S2.** Measurements of protein and nucleic acid release for *P. graveolens* EO NE after 2h Treatment (used at their MICs).

| Bacterial OD_260nm_ OD_280nm_ |
| --- |
| Control *E. coli* 0.365±0.002 0.27±0.003  Control *B. subtilis* 0.380±0.004 0.15±0.002  *E. coli*  1.07±0.052 0.921±0.042  *B. subtilis* 0.687±0.052 0.629±0.034 |

**Table S3.** Measurements of protein and nucleic acid release for *P. graveolens* EO after 2h Treatment (used at their MICs).

| Bacterial OD_260nm_ OD_280nm_ |
| --- |
| Control *E. coli* 0.365±0.002 0.27±0.003  Control *B. subtilis* 0.380±0.004 0.15±0.002  *E. coli*  0.573±0.052 0.700±0.042  *B. subtilis* 0.485±0.052 0.442±0.034 |


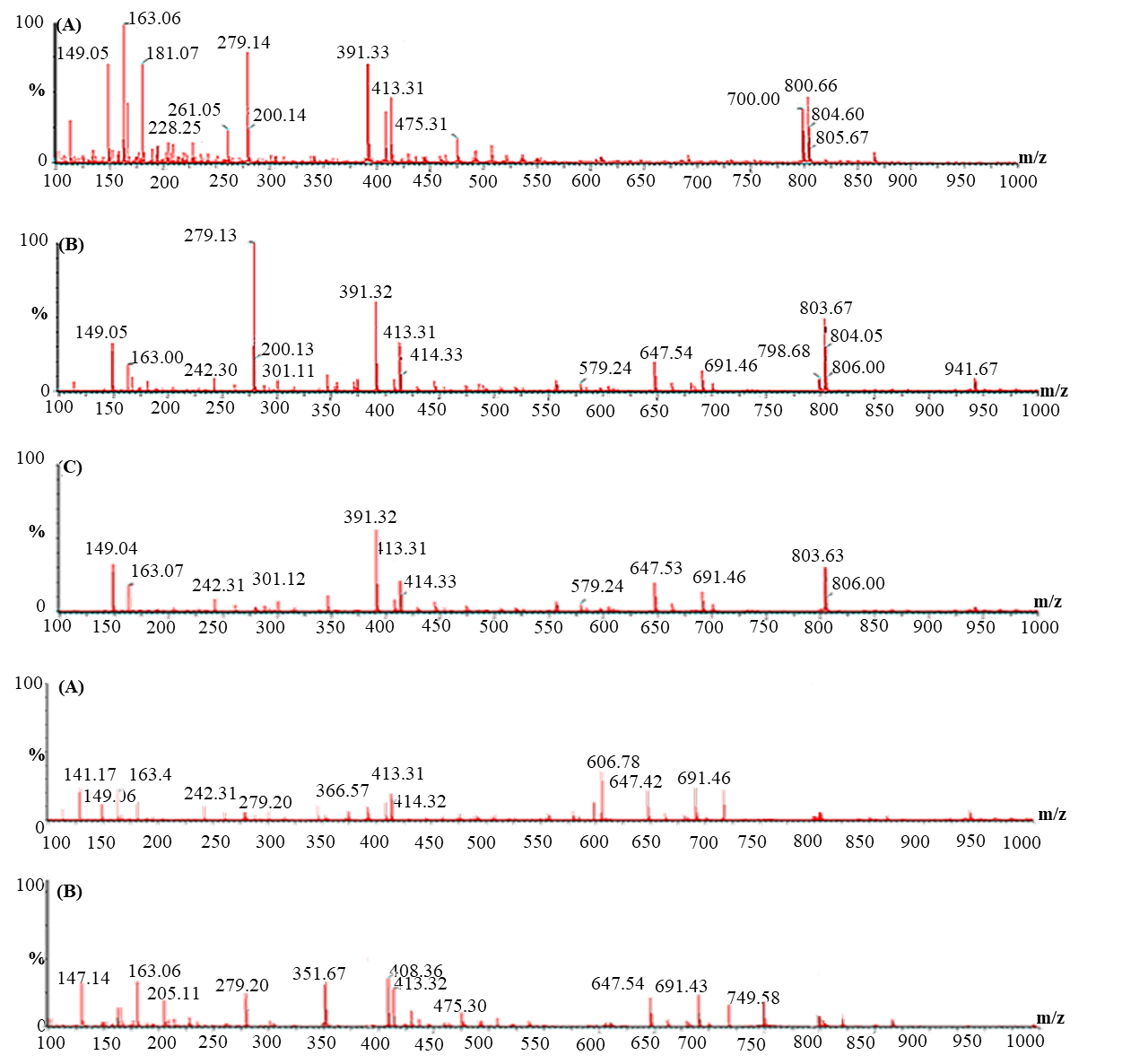


**Fig. S5.** Desorption electrospray ionization (DESI) mass spectrometry (MS) related to *B. subtilis*, (A) intact *B. subtilis* bacteria, (B) *B. subtilis* bacteria treated with nanoemulsion at MIC concentration.


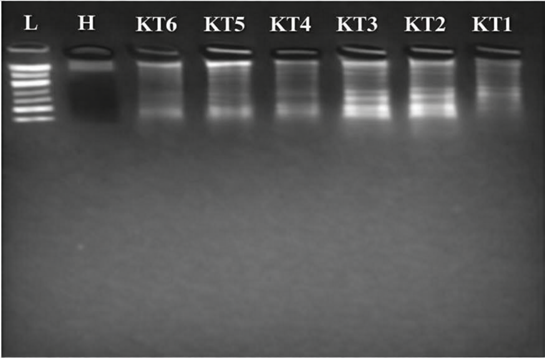


**Fig. S6.** Agarose gel electrophoresis profile of the samples treated with different concentrations of the nanoemulsion. Lane L shows the DNA molecular weight marker (ladder). Lane H represents the H₂O₂-treated control. Lanes KT1–KT6 correspond to samples C1–C6, respectively (KT1 = 25 µg/mL, KT2 = 1 µg/mL, KT3 = 1 µg/mL, KT4 = 6.25 µg/mL, KT5 = 12.5 µg/mL, and KT6 = 3.12 µg/mL).
